# Cheek swabs, SNP chips, and CNVs: Assessing the quality of copy number variant calls generated with subject-collected mail-in buccal brush DNA samples on a high-density genotyping microarray

**DOI:** 10.1186/1471-2350-13-51

**Published:** 2012-06-26

**Authors:** Stephen W Erickson, Stewart L MacLeod, Charlotte A Hobbs

**Affiliations:** 1 Department of Biostatistics, College of Medicine, University of Arkansas for Medical Science, 4301 W. Markham Street, Mail Slot 781, Little Rock, AR, 72205-7199, USA; 2 Department of Pediatrics, College of Medicine, University of Arkansas for Medical Sciences, Arkansas Children’s Hospital Research Institute, Little Rock, AR, USA

**Keywords:** SNPs, Single nucleotide polymorphisms, CNVs, Copy number variants, NBDPS, National Birth Defects Prevention Study, Buccal brush

## Abstract

**Background:**

Multiple investigators have established the feasibility of using buccal brush samples to genotype single nucleotide polymorphisms (SNPs) with high-density genome-wide microarrays, but there is currently no consensus on the accuracy of copy number variants (CNVs) inferred from these data. Regardless of the source of DNA, it is more difficult to detect CNVs than to genotype SNPs using these microarrays, and it therefore remains an open question whether buccal brush samples provide enough high-quality DNA for this purpose.

**Methods:**

To demonstrate the quality of CNV calls generated from DNA extracted from buccal samples, compared to calls generated from blood samples, we evaluated the concordance of calls from individuals who provided both sample types. The Illumina Human660W-Quad BeadChip was used to determine SNPs and CNVs of 39 Arkansas participants in the National Birth Defects Prevention Study (NBDPS), including 16 mother-infant dyads, who provided both whole blood and buccal brush DNA samples.

**Results:**

We observed a 99.9% concordance rate of SNP calls in the 39 blood–buccal pairs. From the same dataset, we performed a similar analysis of CNVs. Each of the 78 samples was independently segmented into regions of like copy number using the Optimal Segmentation algorithm of Golden Helix SNP & Variation Suite 7.

Across 640,663 loci on 22 autosomal chromosomes, segment-mean log *R* ratios had an average correlation of 0.899 between blood-buccal pairs of samples from the same individual, while the average correlation between all possible blood-buccal pairs of samples from unrelated individuals was 0.318. An independent analysis using the QuantiSNP algorithm produced average correlations of 0.943 between blood-buccal pairs from the same individual versus 0.332 between samples from unrelated individuals.

Segment-mean log *R* ratios had an average correlation of 0.539 between mother-offspring dyads of buccal samples, which was not statistically significantly different than the average correlation of 0.526 between mother-offspring dyads of blood samples (*p*=0.302).

**Conclusions:**

We observed performance from the subject-collected mail-in buccal brush samples comparable to that of blood. These results show that such DNA samples can be used for genome-wide scans of both SNPs and CNVs, and that high rates of CNV concordance were achieved whether using a change-point-based algorithm or one based on a hidden Markov model (HMM).

## Background

Multiple investigators have established the feasibility of using buccal brush samples to genotype single nucleotide polymorphisms (SNPs) with high-density genome-wide microarrays [1-3], but there is currently no consensus on the accuracy of copy number variants (CNVs) generated from these DNA samples and genotyping instruments. Regardless of the source of DNA, it is more difficult to detect CNVs than genotype SNPs using these microarrays. There is understandable concern that detecting CNVs is difficult enough using DNA extracted from blood samples, much less using buccal samples, where the amount and quality of DNA might be lower.

Many studies, including the National Birth Defects Prevention Study (NBDPS) [4], rely on subject-collected mail-in DNA samples, because this is a cost-effective way of collecting DNA from a geographically diverse population. Such DNA samples are assumed to have a higher variance in adherence to collection protocols, and furthermore may be subject to suboptimal conditions in transit.

To demonstrate the quality of CNVs generated from these DNA samples and genotyping instruments, we evaluated the concordance of CNV calls from DNA extracted from blood to DNA extracted from subject-collected mail-in buccal brushes (i.e. cheek swabs). The Illumina Human660W-Quad BeadChip was used to determine SNPs and CNVs of 39 Arkansas participants in the NBDPS who had provided both whole blood and buccal brush DNA samples.

## Results

### SNP concordance

Genotype calls across 561,490 SNPs were generated using Illumina’s GenomeStudio software under default settings; genotypes with a GenCall score [5] of 0.15 or lower were considered unreliable and set to no-calls. Among the 39 blood samples, call rates averaged 99.82%, ranging from 99.25% to 99.87%, while call rates for the 39 buccal brush samples averaged 99.81% (99.51% to 99.87%). Concordance rates between blood-buccal pairs of samples averaged 99.92%, ranging from 99.60% to 99.97%. These results confirm that it is possible to get excellent performance in genotyping SNPs from mail-in buccal brush DNA samples on a genome-wide microarray.

### CNV concordance

For our analysis of CNVs, we restricted data to the 22 autosomal chromosomes, because part of our analysis was a comparison of maternal and infant CNVs, and there were both male and female infants in the study sample. In addition to 547,937 SNPs, an additional set of 92,726 non-polymorphic, but copy-number-informative, loci were included in the analysis, for a total of 640,663 loci on 22 autosomal chromosomes. Using the Optimal Segmentation algorithm of SNP & Variation Suite 7 (SVS7; Golden Helix, Bozeman, Montana), each of the 78 samples was independently segmented into regions of common mean. This segmentation was performed on the 78 sequences (one per sample) of 640,663 log *R* ratios, which is defined as the base-2 log-ratio of the sample’s direct intensity (*R*) at the given locus, versus a reference sample [6].

Optimal Segmentation does not explicitly assign copy number state, but instead produces a parsimonious segmentation of the genome in which each locus in a segment shares the same underlying distribution of log *R* ratios. We judged concordance, therefore, by comparing the 78 sequences of segment-mean log *R* ratios, in which individual log *R* ratios are replaced by their corresponding segment mean. Among the 39 blood-buccal pairs of DNA samples from the same individual, segment-mean log *R* ratios had an average Pearson’s correlation coefficient of 0.899, while the average correlation between all possible blood-buccal pairs of unrelated individuals was 0.318.

**Figure 1:**
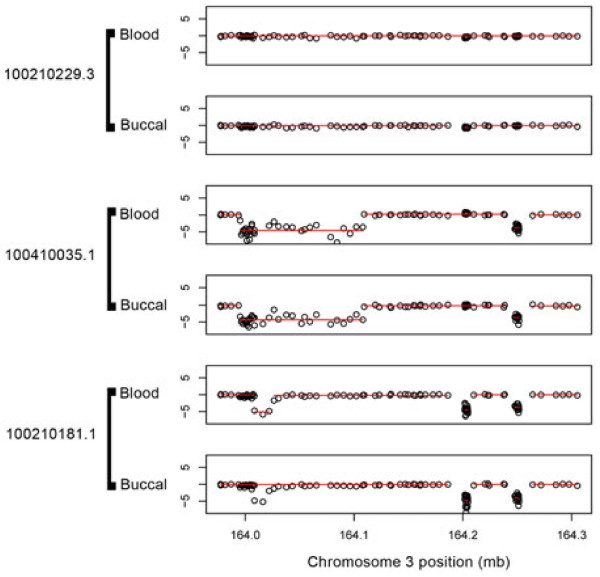
**Illustrative example from a 3kb region of chromosome 3.** The log *R* ratios of three blood-buccal pairs of samples are plotted, with segment means (produced by Optimal Segmentation algorithm) overlaid in red.

As an illustration, Figure 1 shows data from blood-buccal pairs of samples from three unrelated individuals in a 3kb region of chromosome 3. We find multiple deletions in two of these subjects, each of which is detected in both the blood and buccal samples, except for one deletion that spans only three loci and is detected in the blood sample but not in the buccal sample (subject 100210181.1 near 164.0 mb). Figure 1, therefore, illustrates the feasibility of using buccal cell data for CNV detection, but also reveals the challenges involved in detecting CNVs which span only a small number of probed loci.

The average correlation of raw log *R* ratios between the 39 blood-buccal pairs of samples from the same individual was 0.613, while the average correlation of segment-mean log *R* ratios from the same pairs of samples was 0.899 (Table 1). This shows that by combining data across neighboring loci to infer regions of like copy number, the Optimal Segmentation algorithm substantially reduces locus-to-locus variation while still retaining the features (CNVs) shared by the two sequences of data. The average correlation of segment-mean log *R* ratios was 0.526 between mother-offspring dyads of blood and buccal samples, and was 0.318 between the set of all possible blood-buccal pairs from unrelated individuals. The greater correlation between mother-offspring dyads shows that there are heritable CNVs, while the positive correlation between unrelated pairs of samples shows that there are common CNVs shared by unrelated individuals in our population.

**Table 1:** **Average correlation of log *R *ratios between pairs of biological samples**

	**Blood v Buccal**				**Blood**		**Buccal**							
	**Self**	**Mother/Child**	**Unrelated**	**Mother/Child**	**Unrelated**	**Mother/Child**	**Unrelated**							
Raw data	0.613	0.336	0.184	0.369	0.231	0.446	
SVS 7	0.899	0.526	0.318	0.526	0.321	0.539	0.323
QuantiSNP							
BF10	0.933	0.552	0.326	0.549	0.329	0.565	0.334
BF30	0.943	0.550	0.332	0.552	0.335	0.557	0.334
BF50	0.943	0.549	0.335	0.551	0.338	0.557	0.337

Average correlations (Pearson’s) are shown for both raw data and for vectors of segment-mean data under different algorithms. QuantiSNP results are shown for three different log Bayes factor cut-offs.

The correlation between mothers and offspring allows us to compare the performance of buccal and blood samples. We would expect higher quality DNA to yield higher correlations between mothers and offspring, because there would be less measurement error in the resulting data. In our data, segment-mean log *R* ratios had an average correlation of 0.539 between mother-offspring dyads of buccal samples, which was greater than the average correlation of 0.526 between mother-offspring dyads of blood samples, although not significantly so (*p*=0.302). Similarly, correlations averaged 0.323 between all possible unrelated pairs of buccal samples, compared to 0.321 (*p*=0.177) from blood samples. Buccal and blood DNA samples, therefore, performed comparably well in detecting CNVs.

### Comparison of CNV-calling algorithms

QuantiSNP [7] is a Bayesian hidden Markov model (HMM) for inferring CNVs from SNP arrays. We chose QuantiSNP as a contrasting algorithm to Optimal Segmentation because it is HMM-based, is widely used and cited in scientific literature [8-10], and in a recent comparison of seven CNV-calling algorithms [11], outperformed the other six methods in the majority of datasets tested. Each CNV detected by QuantiSNP has a log Bayes factor (LBF) associated with it, indicating the strength of evidence in favor of the CNV versus normal copy number. The QuantiSNP manual [12] recommends an LBF cutoff of at least 30 to obtain low false positive rates; as with any CNV-detecting algorithm, there is a tradeoff between detecting smaller variants (sensitivity) and controlling the rate of false positives (specificity).

An independent analysis using QuantiSNP produced average correlations of 0.943 between blood-buccal pairs versus 0.332 between unrelated pairs, using an LBF cutoff of 30. Table 1 also shows results from applying LBF cutoffs of 10 and 50, suggesting that the correlation in segment-mean log *R* ratio is largely insensitive to the choice of cutoff. Table 2, however, shows that the LBF cutoff does influence the number of CNVs detected, as well as the average size of those CNVs. When using an LBF cutoff of 10, which is the lowest value recommended in the QuantiSNP manual, a significantly higher number of CNVs is detected in the brush data than in the blood data (385.7 per subject vs 314.0, respectively, *p*=0.025). Using an LBF cutoff of 50, however, reduces the average number of CNVs as well as the difference between brush and blood (109.1 per subject vs 103.1, *p*=0.169).

**Table 2:** Characteristics of CNVs detected using QuantiSNP

	**Bayes Factor 10**		**Bayes Factor 30**		**Bayes Factor 50**	
	**Blood**	**Brush**	**Blood**	**Brush**	**Blood**	**Brush**
Mean # CNVS per subject	314.0	385.7	151.5	183.0	103.1	109.1
Size of CNVs (kb)						
Mean	15.9	16.2	19.9	19.0	19.4	19.0
25th percentile	1.0	1.1	1.0	1.1	1.1	1.2
Median	2.1	2.1	1.9	2.1	1.9	2.0
75th percentile	6.0	5.8	4.4	4.8	3.9	4.2
Size of CNVs (probes)						
Mean	15.9	16.6	17.2	18.4	17.9	17.0
25th percentile	12.0	12.0	13.0	14.0	14.0	14.0
Median	16.0	16.0	17.0	17.0	17.0	17.0
75th percentile	18.0	18.0	18.0	18.0	18.0	18.0

**Figure 2:**
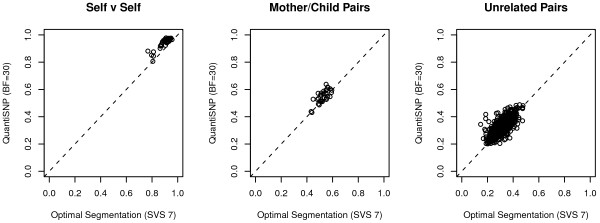
**Correlation between pairs of samples of segment-mean log *R *ratios.** Comparison of results from QuantiSNP (LBF=30) vs. Optimal Segmentation.

Figure 2 compares the blood-buccal correlation of segment-mean log *R* ratios generated by QuantiSNP and SVS Optimal Segmentation. The first panel shows that QuantiSNP yields uniformly higher self-to-self correlations, while the second and third panels show higher average mother-offspring correlations and correlations between unrelated pairs of samples. This may be due to QuantiSNP’s use of B allele frequency data, in addition to log *R* ratio, to infer CNVs, which allows loss of heterozygosity (LOH), for example, to inform CNV calls.

## Discussion

The use of subject-collected mail-in DNA samples exposes a study to an additional risk of non-compliance, namely, the possibility of poor sample collection or handling by study subjects. Out of our initial collection of 46 buccal brush samples that were run on non-defective microarrays, one had a low genotyping call rate that was likely caused by poor DNA quality, and one had a high call rate but was genetically inconsistent with the three other members of its blood/buccal/mother/offspring quartet. This left 44 buccal samples (96%) that had high call rates and the expected genetic relatedness to the rest of their respective quartets. The NBDPS collects DNA from mothers, fathers, and infants, which allows a consistency check of genetic identity. In the buccal brush samples used in this study, total DNA yield ranged from 390 to 11,004 ng and averaged 2,474 ng (Additional file 1: Table 1), while the Illumina BeadChip platform requires 200 ng DNA per sample. Therefore, in the case of defective chips or for purposes of quality assurance, nearly any buccal sample could be re-run on a new chip if desired.

This study investigated the feasibility of using buccal brush DNA samples, instead of blood samples, on a high-density genotyping microarray platform to genotype SNPs and CNVs. Other labs have investigated the accuracy of CNVs detected using microarrays [13], and we recommend replicating any CNV associations using independent samples and alternate lab techniques, such as quantitative real-time PCR (qPCR), regardless of the DNA sample type. Nevertheless, the high correlation of CNV calls within self-to-self blood-buccal pairs of samples, in comparison to substantially lower correlations between unrelated blood-buccal pairs, gives us high confidence that the data, though containing noise and errors, do reflect the actual biological state. Rincon and colleagues [14] have published a comparison of eight matched pairs of canine blood and buccal DNA samples and, similarly, did not find significant differences in marker intensity measurements between the two sources of DNA. Marenne and colleagues, [15] on the other hand, found that human saliva DNA samples did not perform as well as blood in their analysis of CNV. Although these and our current results cannot be extrapolated to all potential sources of DNA, such as urine, hair, or fingernails, they might give investigators with access to these other non-blood DNA samples encouragement to perform similar feasibility studies.

CNV differences between different tissues of the same healthy individuals have been observed [16]. This might be responsible for the consistently higher correlations between blood/blood and buccal/buccal pairs of unrelated individuals, compared with corresponding buccal/blood pairings (Table 1). The fact that these correlations between unrelated individuals were consistently positive is evidence of common CNVs that are shared by multiple individuals in populations.

## Conclusions

We observed performance from the subject-collected mail-in buccal brush samples comparable to that of blood. These results show that such DNA samples can be used for genome-wide scans of both SNPs and CNVs, and that high rates of CNV concordance were achieved whether using a change-point-based algorithm or one based on a hidden Markov model.

## Methods

### Ethics statement

The study was approved by University of Arkansas for Medical Sciences’ Institutional Review Board and the NBDPS with protocol oversight by the Centers for Disease Control and Prevention (CDC) Center for Birth Defects and Developmental Disabilities. All study subjects gave informed written consent. For minors, informed written consent was obtained from their legal guardian.

### Sample collection

In 1997, eight CDC-supported centers for birth defects research were established and began participation in the NBDPS [4,17,18]. A population-based birth defects registry at each site abstracts information on live born or stillborn infants and elective terminations diagnosed with one of 30 major structural malformations. Controls—infants without congenital anomalies—are selected randomly from either birth certificates or hospital records.

Information regarding multiple maternal exposures and lifestyles hypothesized to impact the developing embryo are obtained from all case and control mothers who participate in the NBDPS by a phone interview. Once a mother has completed the interview, a DNA sample-collection kit (with instructions, consent forms, sterile brushes, reimbursement, and return envelope) is mailed to her.

In Arkansas, there is one pediatric tertiary care stand-alone children's hospital – Arkansas Children's Hospital. A subset of Arkansas residents who completed the NBDPS were also included in another study at Arkansas Children's Hospital Research Institute; thus, some Arkansas families who were eligible for both studies provided both blood and buccal cell samples.

### Experimental design

All participants in the NBDPS are asked to submit maternal, paternal, and infant DNA samples using mail-in CytoSoft CYB-1 buccal brush kits [4]. In order to assess the quality of genotypes generated using these samples on the Illumina Human660W-Quad BeadChip, we performed a pilot study of 24 mother-offspring dyads (48 subjects total) for whom whole blood samples were available in addition to buccal brush samples. These 96 DNA samples were prepared and hybridized to BeadChips under standard Illumina protocols [19]. Because the Human660W-Quad handles four DNA samples per chip, each sample of the mother-offspring/blood-buccal quartet of samples was randomly assigned to four different BeadChips, to prevent any possible chip effects from biasing results. After hybridization and scanning, genotypes for 561,490 SNPs were determined for each sample, using Illumina’s proprietary GenCall algorithm under default settings.

Out of the 96 DNA samples, all but six had SNP call rates of 99.1% or higher, with a mean call rate of 99.8%. Four of the six low call rates were caused by a defective BeadChip, which was determined using diagnostic data plots and subsequently confirmed by Illumina. We cannot definitively determine the cause of the remaining two low call rates, which came from one blood and one buccal sample of unrelated individuals on two different BeadChips. Out of the remaining 42 subjects with both high blood and high buccal call rates, 39 had blood-buccal SNP concordance rates greater than 99.9%. Of the three subjects (two infants and one mother) with high call rates and low concordance rates, we were able to determine whether the blood or buccal sample was more likely to contain incorrect DNA, by comparing genotypes to those from the related subject (i.e., mother or child) and assuming Mendelian inheritance.

Based on these comparisons, the three likely mislabeled or miscollected DNA samples were one maternal blood, one infant blood, and one infant buccal sample. Out of the 46 buccal samples that were hybridized to non-defective BeadChips, therefore, one had a low call rate (83%) and one appeared to be either mislabeled or miscollected, based on a high call rate but low concordance with its corresponding blood sample and with the two maternal DNA samples. The other 44 buccal samples (96%) exhibited high call rates and high concordance with their corresponding blood samples, as well as Mendelian consistency with their related samples. There were 39 subjects with high SNP call rates for both blood and buccal, and high SNP concordance rates between the two, and these subjects form the basis for our analysis of CNV. Out of these 39, 32 were comprised of 16 mother-infant dyads.

### DNA collection, extraction, and quantification

Methods for the collection and processing of blood and buccal cells are well established using approved IRB protocols for the DNA Bank for Congenital Malformations and the NBDPS [17]. Samples are logged into electronic inventory at the Hobbs Birth Defects Genomics Laboratory using a bar-code system. DNA is extracted from blood or buccal cell samples by using Pure Gene DNA purification reagents (Qiagen Inc. USA, Valencia, CA) according to the manufacturer’s protocol. Prior to genotyping, genomic DNA are quantified with TaqMan RNaseP Detection Reagents (Applied Biosystems ABI, Foster City, CA) and 200 ng were used for genotyping. Additional file 1: Table 1 shows DNA yields and concentrations for the 78 blood- and buccal-derived samples.

### Generation of SNP and CNV calls

Genotype calls across 561,490 SNPs were generated using Illumina’s GenomeStudio software under default settings; genotypes with a GenCall score of 0.15 or lower were considered unreliable and set to no-calls (Illumina, 2010).

The QuantiSNP manual recommends filtering out CNVs with LBF less than 10 to prevent large numbers of false positive calls, while setting a threshold at 30 or more is recommended to obtain low false positive rates. We therefore computed correlations of segment-mean log *R* ratios under three different LBF thresholds: 10, 30, and 50, representing a range of thresholds that might be used in practice.

## Abbreviations

SNP: Single nucleotide polymorphism; CNVs: Copy number variants; NBDPS: National Birth Defects Prevention Study; IRB: Institutional Review Board; HMM: Hidden Markov model; LBF: Log Bayes factor; LOH: Loss of heterozygosity; CDC: Centers for Disease Control and Prevention.

## Competing interests

The authors declare that they have no competing interests.

## Authors’ contributions

SWE conducted statistical analysis and drafted the manuscript. SLM conducted DNA extraction and genotyping and assisted with the manuscript. CAH participated in the study design and manuscript development. All authors read and approved the final manuscript.

## Pre-publication history

The pre-publication history for this paper can be accessed here:

http://www.biomedcentral.com/1471-2350/13/51/prepub

## Supplementary Material

Additional file 1 **Table S1.**DNA Yields and Concentrations. Click here for file
